# Improving Shear Performance of Precast Concrete Segmental Beams Through Continuous Longitudinal Reinforcements Across Joints

**DOI:** 10.3390/ma18071410

**Published:** 2025-03-22

**Authors:** Yu Zou, Dong Xu

**Affiliations:** 1 School of Architecture and Civil Engineering, Xihua University, Chengdu 610039, China; 2 College of Civil Engineering, Tongji University, Shanghai 200092, China; xu_dong@tongji.edu.cn

**Keywords:** bridge engineering, precast segmental beams, joints, continuous longitudinal reinforcements, shear–bending test, shear

## Abstract

Despite the widespread use of precast concrete segmental bridges (PCSBs), concerns persist regarding their structural reliability, particularly due to the interruption of longitudinal reinforcement at joints. To address this, a novel approach based on the Grid Shear Reinforcement Theory is proposed, featuring precast segmental beams with continuous longitudinal reinforcements across joints. Experimental tests were conducted on one monolithic beam and two segmental beams under combined bending and shear with joint types as the primary variable. Key performance metrics included crack propagation, reinforcement strain, failure modes, stiffness, and load-bearing capacity. Results show that continuous longitudinal reinforcement effectively resists axial tension from shear forces, contributing to shear resistance comparable to stirrups. It also restrains diagonal crack propagation and limits main crack widths, significantly improving shear stiffness. Reinforced joints adhered to the plane section assumption and exhibited monolithic beam behavior throughout loading. These findings highlight the critical role of continuous longitudinal reinforcement in segmental beam joints. The study further compares shear reinforcement design approaches in European Codes, ACI, AASHTO, GB, JTC, and the Grid Shear Reinforcement Theory. Practical construction methods for implementing continuous longitudinal reinforcements are also proposed, offering valuable insights for engineering applications.

## 1. Introduction

Owing to standardization and accelerated manufacturing in the factory, precast concrete segmental bridges (PCSBs) have gained widespread adoption both domestically and internationally [1]. However, the complete disconnection of longitudinal reinforcements at the joints is a notable structural weakness [2,3]. At the same time, the mechanical characteristics of PCSBs are not fully comprehended domestically or internationally, which constitutes a primary reason why mainstream international specifications do not adequately address PCSBs. Exceeding specifications indicates a greater reliance on user interpretation of the technology’s core principles, thereby increasing the risk of unpredictable issues and errors. This situation also hampers the further promotion and development of PCSBs. Consequently, scholars have conducted extensive research on traditional concrete segmental beams both domestically and internationally.

Rabbat et al. [4] tested three precast concrete segmental beams and predicted the flexural bearing capacity using traditional flexural theory. Macgregor et al. [5] verified the stress difference between the internal and external tendons of segmental beams by using three-span continuous beam tests. Aparicio et al. [6,7] conducted bending tests on dry joints of externally prestressed segmental beams, adopted special joint elements to reflect joint cracking, and analyzed externally prestressed segmental beams with a nonlinear beam element model. Huang [8] simulated externally prestressed segmental beams using the solid finite element method. Yuan et al. [9] conducted experiments investigating the ratio of internal and external tendons, assessing their impact on the mechanical properties of segmental beams. They concluded that the ultimate failure of segmental beams typically occurred near the sections where significant changes in bending and shear forces occurred. Jiang et al. [10,11] performed tests on 14 externally prestressed concrete beams, verified the influence of the number of segments on the bending properties, and proposed a calculation method. Moustafa [12] conducted shear tests on externally prestressed concrete segmental beams with epoxied joints. Ramirez [13] performed shear–bending tests to show the effects of dry joints and epoxied joints on failure modes and the bearing capacity of segmental beams. Turmo et al. [14] conducted experiments on externally prestressed segmental beams with dry joints. Their tests revealed the stress mechanisms of segmental beams subjected to combined bending and shear forces. They observed that the stirrups placed on both sides of the joint made only a minor contribution to shear resistance. Li’s [15] research results demonstrated the function of the internally prestressed tendons, revealed the failure mode of the joint near the loading point, and provided a calculation method for the bearing capacity of this failure mode according to the equilibrium equation. Through the tests, the simplified calculation equation of the stress increment of the externally prestressed tendons and the simplified calculation equation of the secondary effect of the externally prestressed tendons were obtained. Brenkus et al. [16] conducted an experimental study on a segmental beam with a unique shape. Takebayashi et al. [17] carried out a bending test on a 45 m full-size precast segmental dry-joint beam. The results indicated that at the designed load, the dry joint did not experience slip, and the structure did not incur damage to the diagonal section. Hindi’s [18] test results showed that the strength and ductility of precast segmental bonded tendon beams were higher than those of precast segmental unbonded tendon beams. Sivaleepunth et al. [19] conducted experimental studies on the segment lengths, joint types (flat joint, key joint), and the normal stress levels of segmental beams. The test results indicated that the crack propagation mode of segmental beams was related to the normal stress, the shear strength was less affected by segment lengths, and the shear force near the support point could be transferred through concrete keys. Halder et al. [20] argued that third-order two-variable response surfaces can accurately predict increases in the stress and total stress of tendons in externally prestressed precast segmental concrete bridges. Tran et al. [21,22] reported that the ultimate stress of unbonded FRP tendons in prestressed precast segmental concrete bridges should be limited to 75% of the tensile strength of the tendon in design. Le et al. [23,24] found that the loading type, concrete strength, and the number of joints had insignificant effects on the flexural performance of the structure. Further, they expressed the belief that both AASTHO-1999 [25] and ACI 440.4R-04 [26] could effectively predict tendon stress; however, the accuracy was significantly reduced for cases with unbonded tendons.

To summarize, the simplified calculation of segmental beams employs the section method, aligning with specifications but lacking an adequate foundation. The elastoplastic finite element method or comprehensive test methods account for the entire loading process, including internal force redistribution, diverging from the sectional method specified. Consequently, their safety assessment differs from the sectional method, with much of the research focusing on bending issues. The shear performance of segmental beams is more complex. Previous research indicates that increasing internal tendons enhances the mechanical characteristics of segmental beams, whereas stirrups near the joint do not contribute to shear bearing capacity. This phenomenon primarily stems from the discontinuity of longitudinal reinforcement at the joint, limiting shear stress transfer to the upper slab concrete alone. To address this weakness, the present research team proposed a novel type of precast segmental beam featuring continuous longitudinal reinforcement at the joint. Yet, research into the shear mechanism of this particular segmental beam type remains lacking both domestically and internationally.

In the present study, it was observed that segmental beams consist fundamentally of plain concrete segments and reinforced concrete segments. The failure modes of traditional concrete segmental beams were also summarized. The concept of continuous longitudinal reinforcement at the joint of segmental beams was proposed based on the Grid Shear Reinforcement Theory from the point of “eliminating” the joint in terms of force transmission. To validate the beneficial impact of longitudinal reinforcement at joints, three test beams were designed: a monolithic beam, a segmental beam with continuous longitudinal reinforcement, and a traditional segmental beam. The test parameters included different joint types, and the mechanical properties of the specimens under shear–bending coupling loading were compared. Key research objectives included crack propagation, reinforcement strain, failure mode, structural deformation, and shear capacity assessment. The shear mechanism of the continuous longitudinal reinforcement segmental beams was revealed. The essential role of continuous longitudinal reinforcement at segmental beam joints was further confirmed. Finally, the design methods of the structural shear reinforcement in the European Code [27], the American ACI [28]/AASHTO [25,29], the Chinese GB [30]/JTG [31] Code, and the Grid Shear Reinforcement Theory [32,33] were compared and analyzed, and construction methods for continuous longitudinal reinforcement segmental beams were introduced.

## 2. The Fundamental Nature and Failure Mode of Segmental Beams

**Figure 1:**
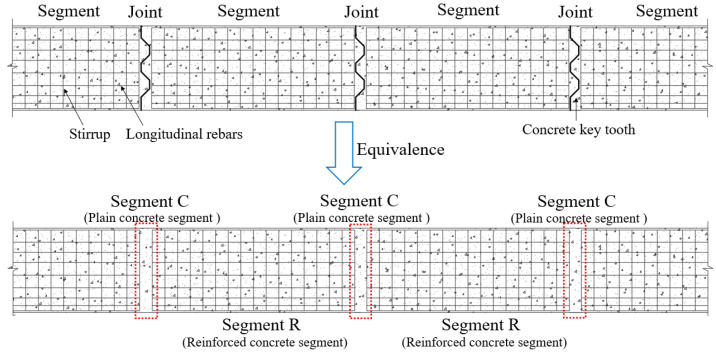
The fundamental nature of segment beams.

The fundamental nature of PCSBs can be understood as a reinforced concrete structure consisting of several plain concrete segments interspersed throughout. These segments include the unreinforced plain concrete segment (Segment C, that is, the joints), formed by discontinuous longitudinal reinforcement and spacer stirrups, and the reinforced concrete segment (Segment R) featuring continuous longitudinal reinforcement, as illustrated in Figure 1.

**Figure 2:**
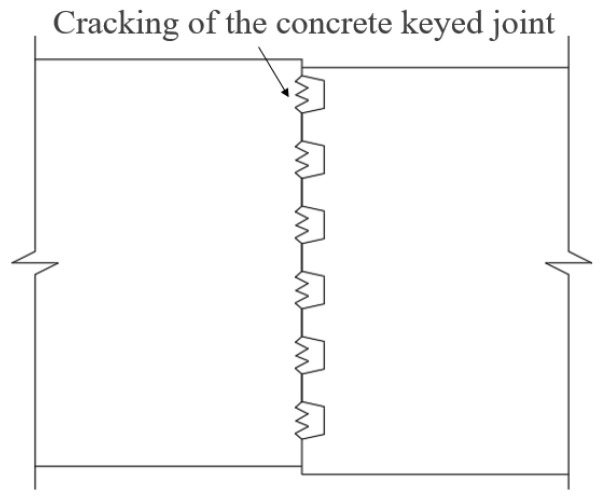
Crack patterns near the joint.

The joint (Segment C) embodies both the characteristic features and vulnerabilities of precast segmental beams, with its mechanical performance crucial to the overall structure. Numerous research findings indicate that crack propagation in precast segmental structures primarily occurs near the joint [5], as shown in Figure 2.

## 3. Design of Continuous Reinforcement Crossing the Joints

**Figure 3:**
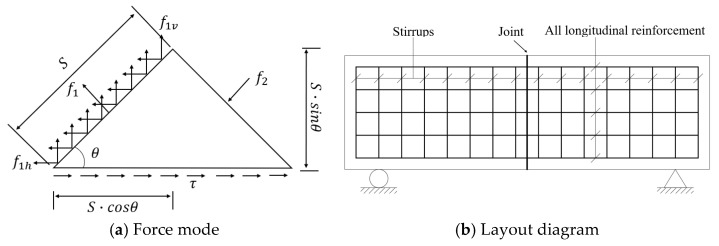
Orthogonal grid shear reinforcement.

Based on the Grid Shear Reinforcement Theory [33], when diagonal cracks develop in concrete, the principal tensile stress initially carried by the concrete is transferred to the steel reinforcement. In segmental beams, vertical stirrups are responsible for resisting the vertical component of the principal tensile stress; however, they cannot balance the horizontal component, resulting in the formation of wide cracks at the joints. To address this limitation, it is essential to establish a force mechanism capable of bearing the horizontal component of the principal tensile stress. A direct and effective solution is to configure longitudinal web reinforcement at the joint to function as shear reinforcement, analogous to the role of stirrups. This concept is illustrated in Figure 3a.

In engineering practice, the combination of longitudinal web reinforcement and vertical stirrups at joints forms what is known as orthogonal grid shear reinforcement, as depicted in Figure 3b. This configuration not only enhances the shear resistance of the joints but also mitigates crack propagation, thereby improving the overall structural performance of segmental beams. In traditional reinforcement methods, horizontal web reinforcement is typically arranged as structural reinforcement without explicit consideration in shear design. In contrast, the Grid Shear Reinforcement Theory treats horizontal web reinforcement as shear reinforcement, similar to stirrups, requiring explicit calculation and design for shear resistance.

In the infinitesimal body mechanical model (see Figure 3a), the principal tensile stress ($f_{1}$, MPa) decomposes in two directions: vertical ($f_{1v}$, MPa) and horizontal ($f_{1h}$, MPa). The principal tensile stress in the vertical component of $f_{1}$ is borne by the stirrup, while the horizontal component is borne by the longitudinal web reinforcement. After the cracking of the beam, the principal tensile stress is shared by the stirrup and longitudinal web reinforcement, which is equivalent to the stress state before the cracking of the beam. The principal tensile stress is replaced by the resultant force generated by the stirrup and longitudinal web reinforcement, and the whole infinitesimal body remains in equilibrium.

**Figure 4:**
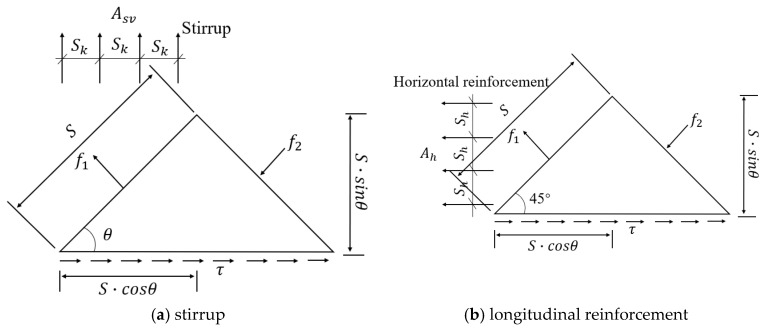
Grid shear reinforcement calculation diagram.

Figure 4 shows the design and calculation diagram of grid shear reinforcement. The crack length of the infinitesimal body is S (mm), and the inclination angle of the principal compressive stress of concrete is θ (°). After cracking, the horizontal and vertical components of the principal tensile stress are borne by the longitudinal reinforcement and stirrup, respectively, and the corresponding equilibrium equation is as follows:

(1) f1·S·bw·cosθ=fsv·Asv·S·cosθSk

(2) f1·S·bw·sinθ=fsh·Ash·S·sinθSh

After simplifying the aforementioned formula, the ratio of the longitudinal reinforcement and the stirrup is obtained as follows:

(3) $$\frac{A_{sv}}{S_{k}}=\frac{f_{1}\cdot b_{w}}{f_{sv}}$$

(4) $$\frac{A_{sh}}{S_{h}}=\frac{f_{1}\cdot b_{w}}{f_{sh}}$$

where $f_{sh}$ is the yield strength of the longitudinal web reinforcement, MPa; $f_{sv}$ is the yield strength of the stirrup, MPa; $A_{sh}$ is the area of the longitudinal web reinforcement, mm^2^; $A_{sv}$ is the area of the stirrup, mm^2^; $b_{w}$ is the width of the web, mm; $f_{1}$ is the principal tensile stress, MPa; $f_{2}$ is the principal compressive stress, MPa.

## 4. Experimental Program

### 4.1. Test Specimens Design

#### 4.1.1. Section Design

**Figure 5:**
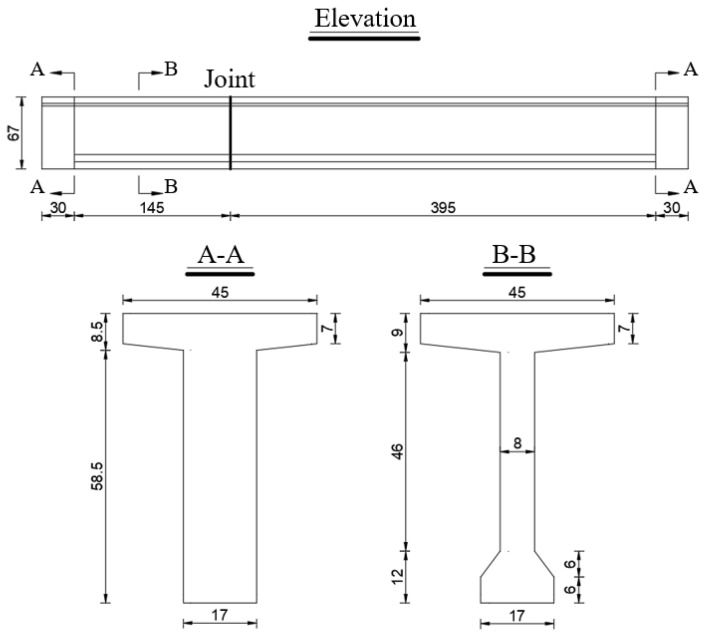
Section size of test beams (unit: cm).

The dimensions of the cross-section, as well as the configurations of the reinforcement and prestressing tendons, were designed based on previous studies by Zou [34], Li [15], Aparicio [7], and Jiang [35]. Modifications were implemented to align with the objectives of this research and the constraints of the laboratory environment. A T-shaped cross-section was selected as the standard design, with a uniform web thickness of 8 cm. At the support sections, the web width was increased to 17 cm to match the dimensions of the bottom plates. The joint positions and sectional details are illustrated in Figure 5.

#### 4.1.2. Reinforcement Design

**Figure 6:**
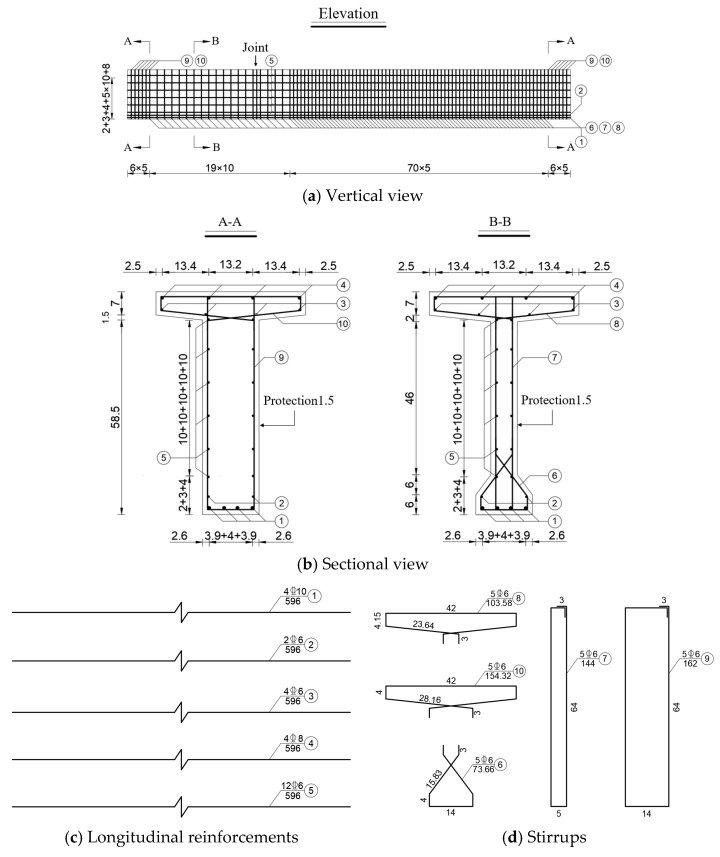
Reinforcement layout [36] (unit: cm).

The reinforcement configuration for the test beams was designed based on Equations (1)–(4). HRB400 steel bars were utilized for the stirrups, as well as the longitudinal reinforcements in the web, top plate, and bottom plate. The stirrups were designed as double-legged with a standard spacing of 100 mm. However, to avoid localized failure at the loading and support regions and to reduce shear damage in the non-jointed shear span zone, the stirrup spacing in these critical areas was decreased to 50 mm. Additionally, longitudinal web reinforcements were installed on both sides of the web at 100 mm intervals. The detailed reinforcement arrangement is depicted in Figure 6a–d.

#### 4.1.3. Tendon Arrangement

**Figure 7:**
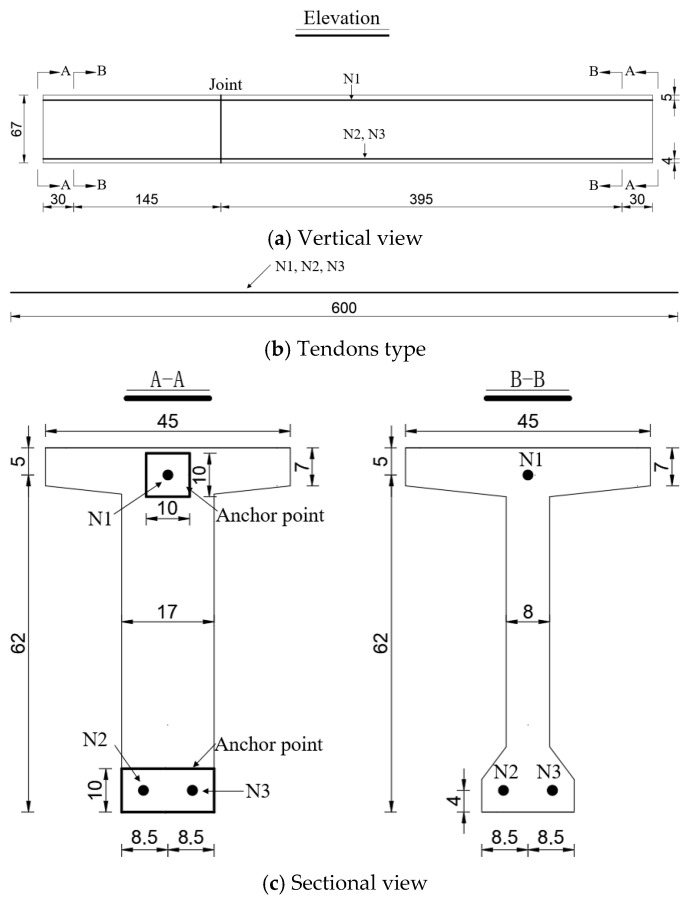
Tendon layout (unit: cm).

Internally bonded tendons were employed in the test beams, with the layout of the prestressing tendons detailed in Figure 7a–c.

#### 4.1.4. Parameter Design

**Figure 8:**
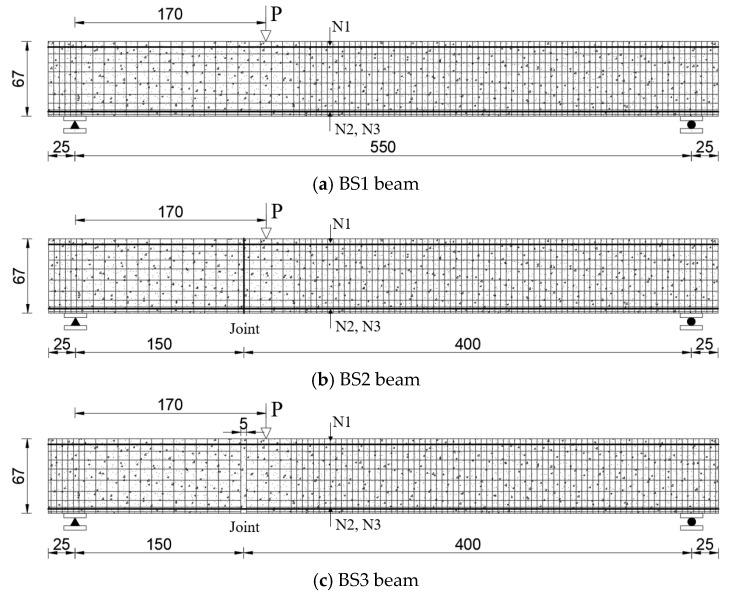
Schematic diagrams of the test beams [36] (unit: cm).

Three test beams were designed for the experiment, focusing on the impact of joint types on the shear behavior of segmental beams. All specimens shared identical cross-sectional dimensions, reinforcement ratios, and shear span ratios (λ = 2.66). Beam BS1, illustrated in Figure 8a, was a monolithic beam. Beam BS2, shown in Figure 8b, represented a segmental beam with continuous longitudinal reinforcement at the joint, constructed using a two-stage concrete pouring process. Beam BS3, depicted in Figure 8c, simulated conventional segmental beams with a keyed joint, featuring discontinuous longitudinal reinforcement and a single-stage concrete pouring process. The structural design of the beams adhered to the GB 50010-2010 [30] code, and their detailed parameters are provided in Table 1.

### 4.2. Materials

**Figure 9:**
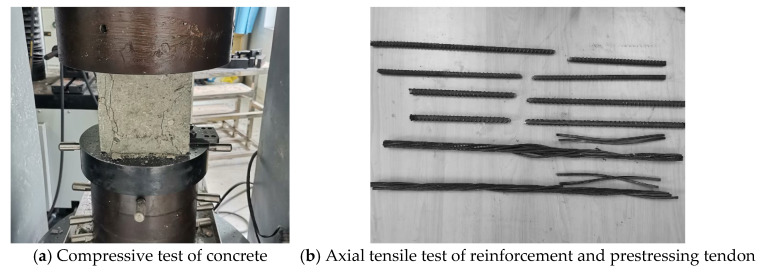
Material characteristics test.

The test beams were constructed using C40 commercial concrete, with the same batch employed for both the sample blocks and the beam casting. The material property testing procedure is illustrated in Figure 9a, and the corresponding strength values are provided in Table 2. Additionally, the same batch of reinforcement was utilized for both the mechanical property tests and the test beams. The testing process for the reinforcement is depicted in Figure 9b, while the mechanical properties of each steel bar are detailed in Table 3.

The prestressing tendons had a diameter of 15.2 mm. The same batch of tendons was applied in both the mechanical property test and for the test beams. The material characteristics test procedure is illustrated in Figure 9b, and the mechanical property parameters of the tendons are presented in Table 3. During the stretching of the prestressing tendons, pressure sensors were positioned at the beam’s end. The prestressing system made use of internally bonded tendons, and the effective prestressing values of each test beam during the loading process are shown in Table 4.

### 4.3. Test Setup

#### 4.3.1. Support and Loading Points Layout

**Figure 10:**
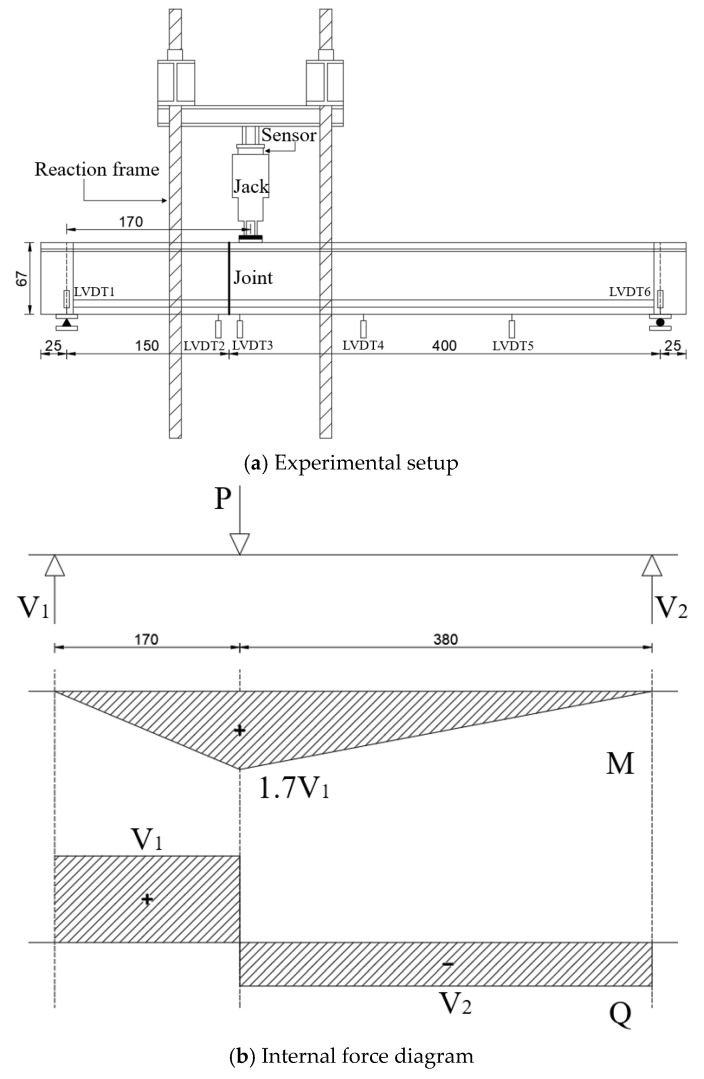
Supports and loading layout (unit: cm).

The shear-bending tests were performed using a single-point loading method, with a consistent shear span of *a* = 1700 mm for all specimens. The loading configuration and the arrangement of displacement meters are illustrated in Figure 10a, while the distribution of internal forces is depicted in Figure 10b.

#### 4.3.2. Measure Point Arrangement

**Figure 11:**
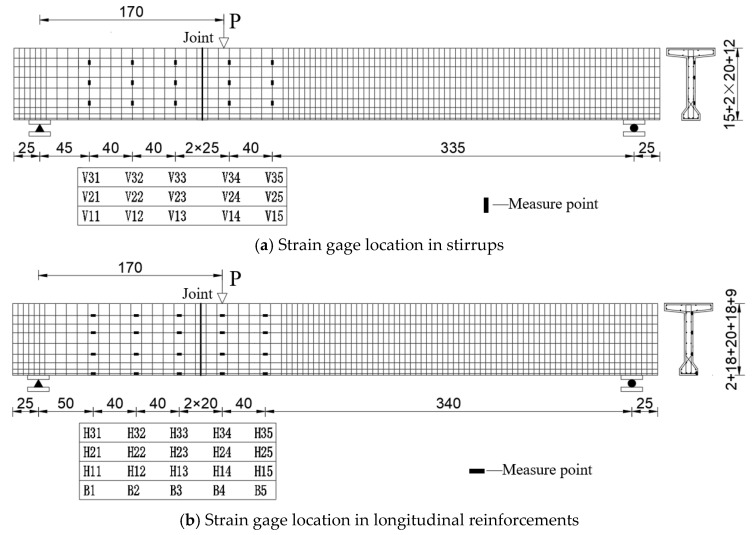
Strain gage arrangement (unit: cm).

The reinforcement strain gages were primarily positioned at the section of the loading point, at 1/2 of the beam height section from the support, and at 1 times the beam height section from the joint. The parameters of the strain gage are shown in Table 5. The numbers of strain gages are numbered from left to right and from bottom to top in Figure 11. The letter H denotes the longitudinal reinforcements of the web; V denotes the stirrups; B denotes the longitudinal reinforcements of the bottom plate; and BS denotes the test beam. For example, BS1-V11 represents the strain gage at the No. 1 position in the first row of the stirrups of BS1; BS1-H11 represents the strain gage at the No. 1 position in the first row of the longitudinal reinforcements of the web of BS1; and BS-B1 represents the strain gage at the No. 1 position of the longitudinal reinforcements of the bottom plate of BS1.

#### 4.3.3. Loading Scheme

The test employed a monotonic loading mode. A ZFXIMP-1B dynamic data acquisition system was utilized to collect vertical loading force and displacement data. The loading force and measurement data were recorded at various load levels, while test phenomena were observed, and crack development was marked during loading. Initially, each load level was set at 10 kN until the specimen approached cracking. After concrete cracking occurred, subsequent load levels were reduced to 5 kN. Following reinforcement yielding, displacement loading was applied at a continuous rate of 0.1 mm·min^−1^ without interruption.

**Figure 12:**
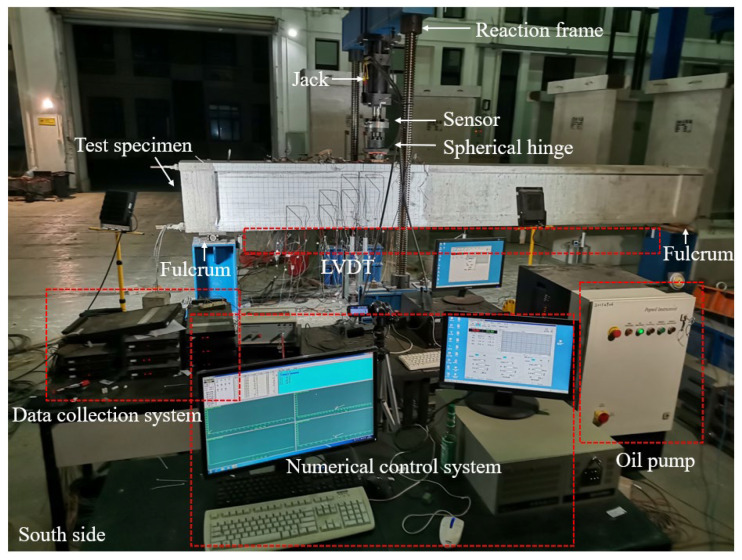
Loading diagram (unit: cm).

The loading system is illustrated in Figure 12. Displacement data were collected using linear variable differential transformers (LVDTs) installed at critical locations, ensuring accurate measurement of deformation throughout the loading process. Force data were recorded simultaneously at a sampling rate of 1 Hz, providing a comprehensive dataset for analyzing the structural response. The beams were prestressed using internal bonded tendons, and no significant variation in the prestressing force was observed during the test.

## 5. Structural Response and Comparison

Based on the test results, the mechanical properties of the monolithic beam, the continuous longitudinal reinforcement segmental beam, and the traditional segmental beam were compared and analyzed in terms of the mechanical behaviors such as crack propagation, reinforcement strain, structure deformation, failure mode, structure stiffness, and shear capacity.

### 5.1. Crack Propagation

**Figure 13:**
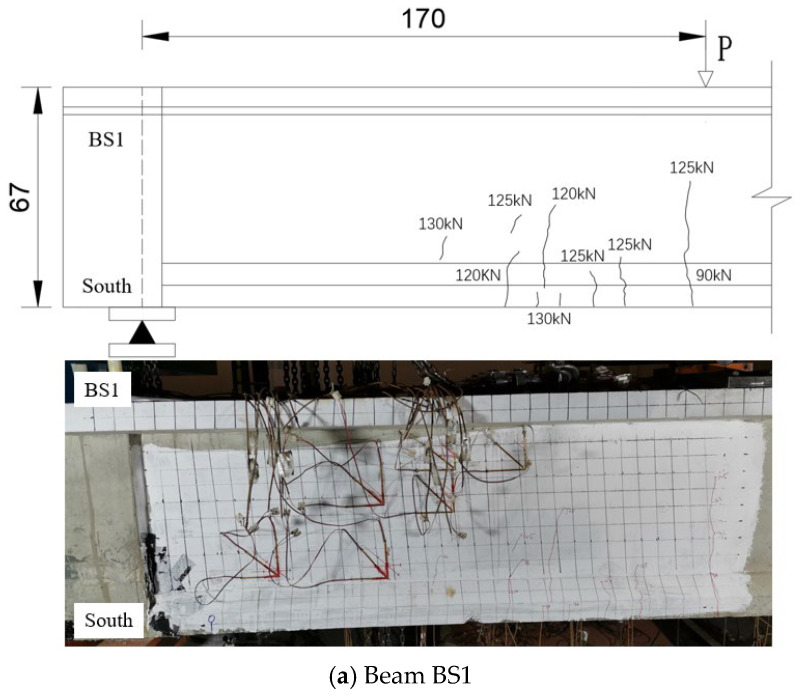

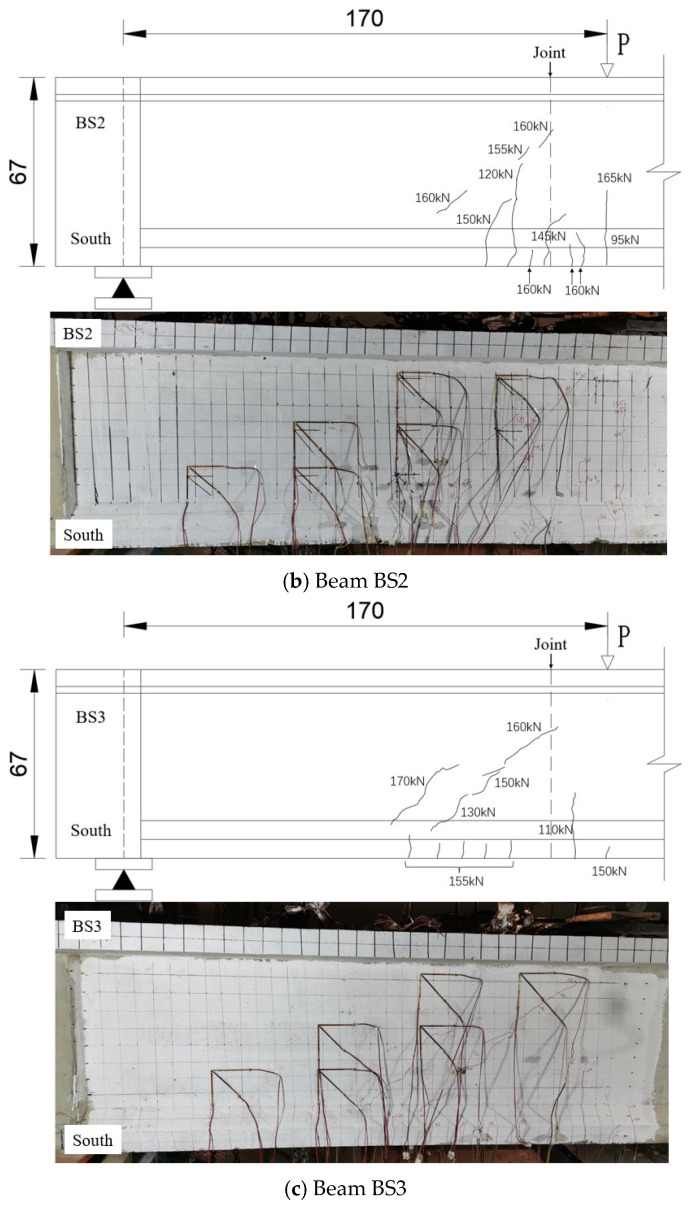
Incipient cracks of beams BS1~BS3 (unit: cm).

Throughout the testing process, comprehensive data on crack propagation were recorded, including the position, length, width, and associated loading force for each crack. In beams BS1 to BS3, the formation of vertical bending cracks and diagonal shear cracks in the web was primarily observed. The first three vertical bending cracks and the first three diagonal shear cracks were designated as incipient cracks, which formed the basis for comparing and analyzing the mechanical behavior of each beam. The incipient vertical bending cracks and diagonal shear cracks for beams BS1-BS3 are detailed in Table 6 and Table 7, respectively, while Figure 13 illustrates the incipient crack patterns.

As indicated in Table 6 and Figure 13, initial vertical bending cracks developed in beams BS1 to BS3. For beams BS1 and BS2, these cracks were located at the bottom edge directly beneath the loading point. In contrast, the initial bending crack in beam BS3 appeared 10 cm away from the loading point on the bottom edge. The initial crack lengths varied between 20 and 30 mm, with widths ranging from 0.050 to 0.060 mm. The corresponding loading forces at the onset of initial bending cracks ranged from 90 to 110 kN, while the forces at the appearance of incipient bending cracks spanned from 90 to 155 kN.

As depicted in Table 7 and Figure 13, the web shear diagonal cracks appeared later than the bending cracks in both monolithic and segmental beams, but there was a minimal difference in loading force. Except for the third diagonal crack of BS1, the initial diagonal cracks of beams BS1~BS3 were all located within 1 times the beam height from the loading point. The width of these diagonal cracks ranged from 0.065 to 0.179 mm, and their length varied between 50 and 200 mm. The loading force at which each specimen exhibited the initial diagonal crack ranged from 120 to 130 kN, and from 120 to 160 kN when the incipient diagonal crack appeared.

An observation can be made from the test results that the loading force, crack location, crack length, and crack width of BS1~BS3 were considerably similar when the incipient cracks appeared. It can be concluded that the mechanical properties of segmental beams (both continuous longitudinal reinforcement segmental beams and traditional segmental beams) are essentially similar to those of monolithic beams at the incipient stage when cracks first appear.

**Figure 14:**
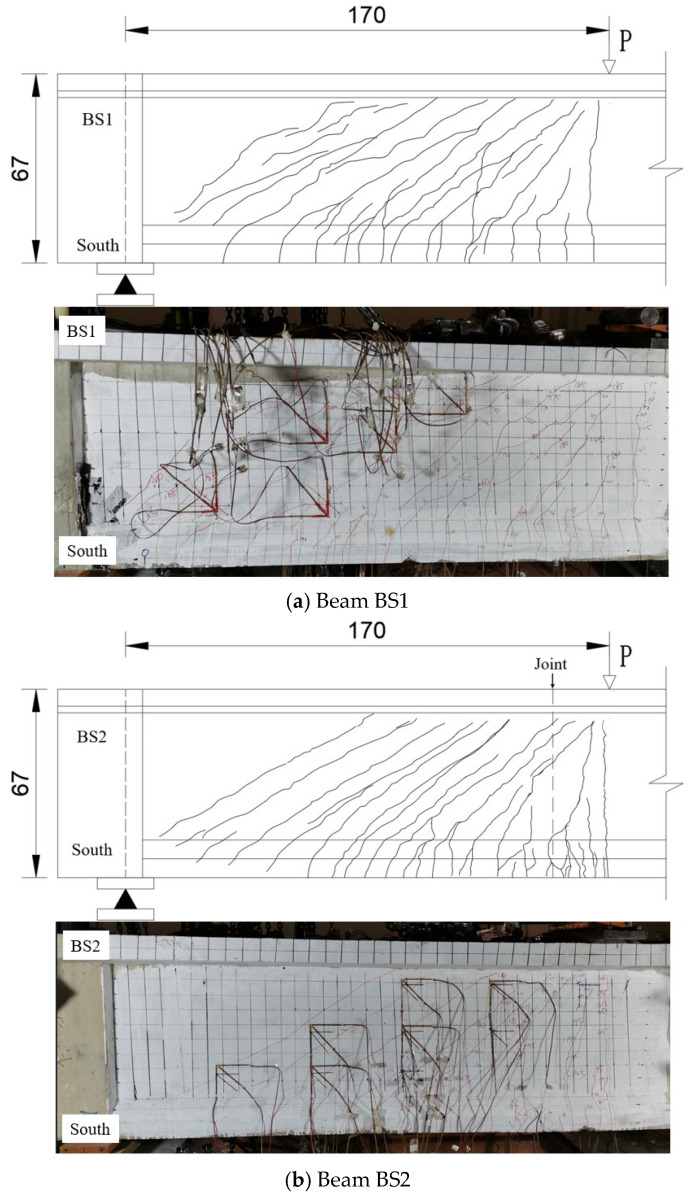

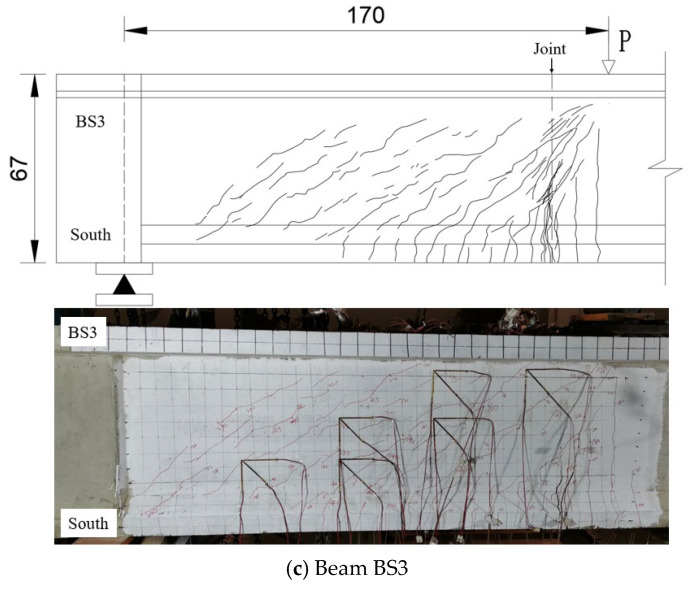
Fully developed crack of beams BS1~BS3 (unit: cm).

Figure 14 illustrates the crack patterns of beams BS1 to BS3 after complete crack development. During the initial loading phase of beam BS1, cracks mainly formed in the web region close to the loading point. With increasing load, the crack propagation area progressively moved toward the web near the support. Concurrently, the crack angles gradually decreased, and the cracks fully extended within the shear span, leading to the formation of a principal crack between the loading and support points.

Similarly, beam BS2 exhibited a crack development process that transitioned from the loading point area to the bearing point area. Diagonal cracks in the web fully developed within the shear span, and a main crack formed between the loading and bearing points, displaying a propagation pattern akin to that of beam BS1.

For beam BS3, cracks initially concentrated within a distance equal to the beam height from the loading point. As the load increased, multiple thin diagonal cracks appeared near the support point, though they remained unconnected. Simultaneously, the crack width at the joint position expanded more rapidly, eventually resulting in a principal crack directed toward the loading point. This crack ultimately developed into an inverted V-shaped opening.

Based on the described test results, the crack development characteristics varied among beams BS1 (monolithic beam), BS2 (continuous longitudinal reinforcement segmental beam), and BS3 (traditional segmental beam). Initially, all beams exhibited similar crack propagation states, suggesting comparable mechanical behavior. However, as loading continued towards failure, distinct differences emerged. BS1 displayed well-developed web shear diagonal cracks distributed across its entire web shear span. BS2 showed fully developed web shear diagonal cracks passing through the joint section, akin to BS1. In contrast, BS3 exhibited concentrated crack development at the joint, resulting in a notably widened main crack that formed an inverted V-shaped opening upon failure. These results underscore the effectiveness of continuous longitudinal reinforcement at the joint, as seen in BS2, in mitigating the opening and propagation of the main crack compared to traditional segmental beams such as BS3.

### 5.2. Reinforcement Strain Variation

#### 5.2.1. Web Longitudinal Reinforcement

**Figure 15:**
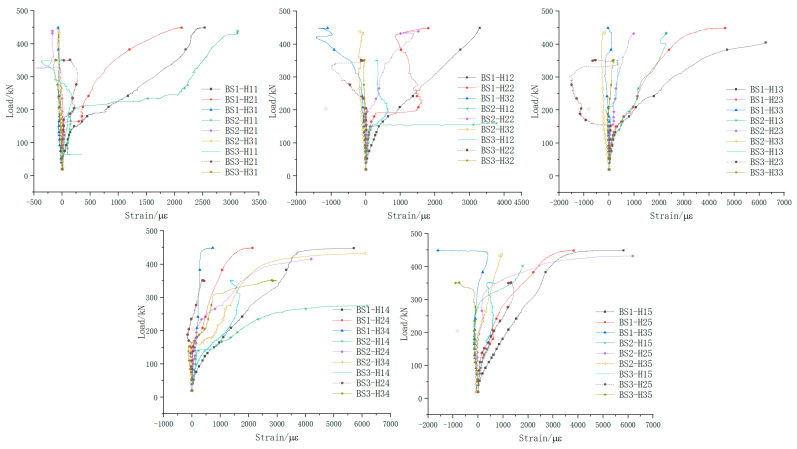
Comparison of web longitudinal reinforcement load–strain curves.

Figure 15 shows the comparison of load–strain curves of web longitudinal reinforcement for beams BS1 to BS3. The vertical axis represents the applied load (kN), while the horizontal axis indicates the strain (με) of the reinforcement.

During the failure of beam BS1, tension strains at measuring points H11# to H15#, H23#, and H25# all reached the yield strain, with points H21#, H22#, and H24# considerably close to yielding. Strain changes at points H31# to H35# were minimal, while compressive strains at H32# and H35# were relatively high.

When beam BS2 was damaged, tension strains at measuring points H11# to H14#, H24#, H25#, and H34# all reached the yield strain. Measuring points H15# and H22# had tension strains very close to the yield strain. Measuring points H21#, H23#, H31#, H32#, H33#, and H35# showed minimal strain changes. Pressure strains appeared at measuring points H31# and H33#.

When beam BS3 failed, the main crack passed through the web longitudinal reinforcement at measuring point H34#. Among all the web longitudinal reinforcement measuring points, only H34# reached the yield strain in tension, while the other points at H11# to H15#, H21# to H25#, H31# to H33#, and H35# did not reach the tensile yield strain.

**Figure 16:**
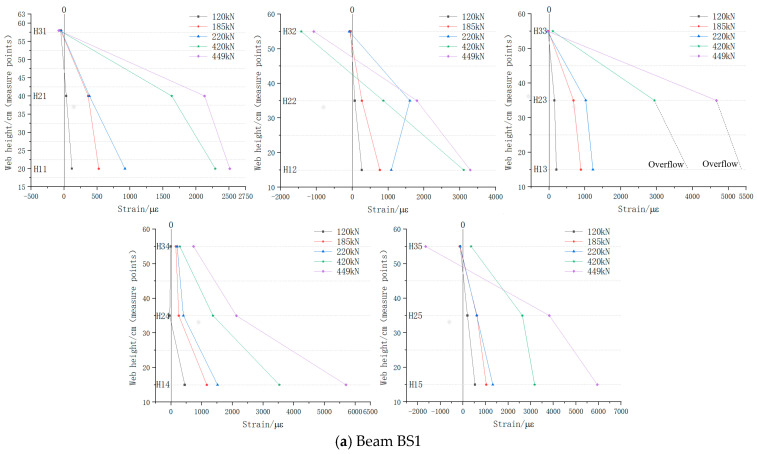

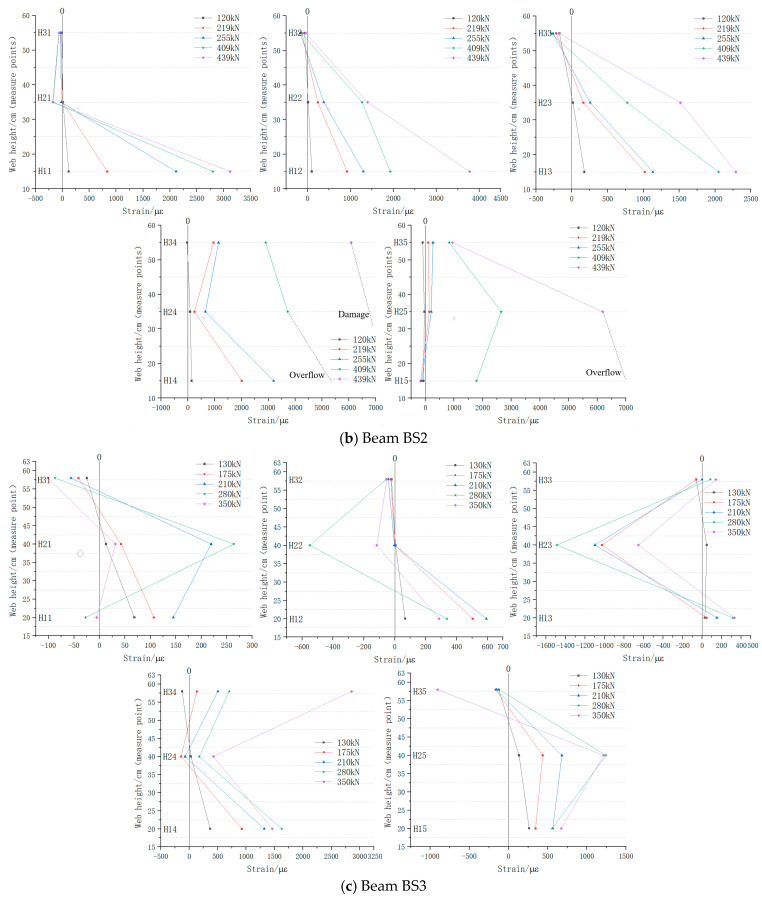
Longitudinal reinforcement strain distribution along web height.

Figure 16 illustrates the longitudinal reinforcement strain distribution along the web height of each specimen. The vertical axis represents the distance (cm) of the strain gauge position from the bottom edge of the web, while the horizontal axis represents the corresponding strain values (με). In beams BS1 and BS2, from initial cracking to specimen failure, the reinforcement strain in each section varied linearly along the beam height, transitioning from tensile strain at the bottom edge of the web to compressive strain at the top edge. However, in BS3, while the reinforcement strain initially varied linearly along the beam height when the initial crack appeared, subsequent loading caused irregular changes in longitudinal reinforcement strain across the web sections. This deviation indicated that the structure no longer satisfied the plane section assumption as loading continued.

According to the test outcomes, it is clear that in both the monolithic beam and the segmental beam with continuous longitudinal reinforcement, the web longitudinal reinforcement functioned effectively in dealing with the axial tension forces induced by shear. These forces were efficiently transmitted along the longitudinal axis of the main beam. This reinforcement mechanism mirrored the role of stirrups in resisting shear by managing the horizontal component of the principal tensile stress. In contrast, the traditional segmental beam, with its discontinuous web longitudinal reinforcement at the joint, struggled to transfer shear-induced axial tension forces effectively. This limitation resulted in the joint forming a V-shaped opening and insufficient development of web diagonal cracks within the shear span area. These findings underscore the critical role of the continuous longitudinal reinforcement at joints in optimizing the shear performance of segmental beams. They ensure robust transfer of shear forces, mitigate structural vulnerabilities such as joint openings, and promote the adequate development of web diagonal cracks essential for structural integrity under shear loading.

#### 5.2.2. Stirrup

**Figure 17:**
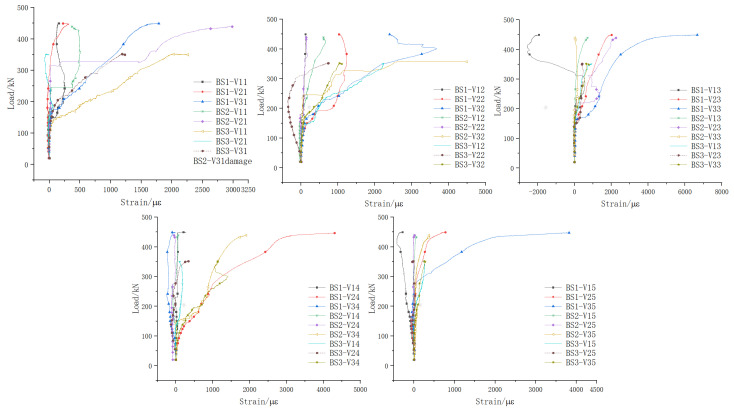
Stirrup load–strain curves.

Figure 17 shows a comparison of the stirrup load–strain curves for beams BS1~BS3. The vertical axis represents the applied load (kN), while the horizontal axis indicates the strain (με) of the reinforcement. In BS1, sections 1# to 5# of the stirrup, except for section 1#, reached at least one measuring point in sections 2# to 5# that attained tensile yield strain. Similarly, in BS2, sections 1# to 5# of the stirrup, except for section 5#, had at least one measuring point in sections 1# to 4# reaching tensile yield strain. Conversely, as the primary crack at the joint of BS3 gradually widened, the tensile strain in sections 4# and 5# of the stirrup diminished. This led to a further relaxation of the structural stress on either side of the crack. sections 1# and 2#, located farther from the joint, functioned as the mechanism for transferring shear force, resulting in their stirrup strain approaching the tensile yield strain.

From the described test results, it can be concluded that the stirrup of the monolithic beam and the continuous longitudinal reinforcement segmental beam could effectively bear the vertical component generated by the principal tensile stress when the specimen was damaged and fully utilize the role of the shear reinforcement. Contrastingly, the joint section of the traditional segmental beam lacked the confinement provided by longitudinal reinforcement as the joint opened further. This led to the full release of structural stress on both sides of the joint section, where shear stress could only transfer through the concrete in the shear compression zone at the upper edge of the joint and the prestressing tendons. Consequently, the shear resistance effectiveness of stirrups near the joint gradually diminished.

#### 5.2.3. Bottom Longitudinal Reinforcement

**Figure 18:**
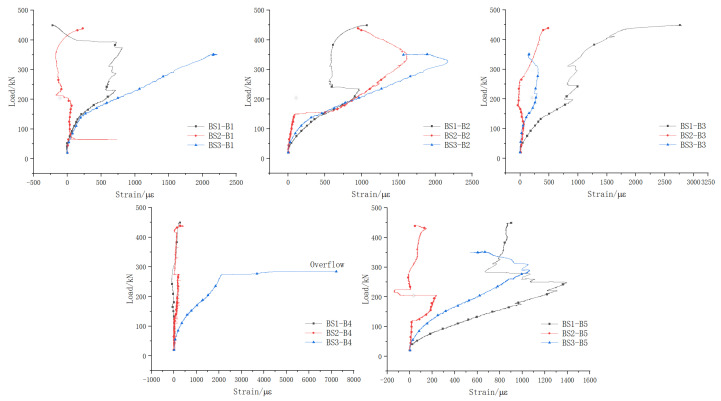
Comparison of bottom longitudinal reinforcement load–strain curves.

Figure 18 shows a comparison of the load–strain curves of longitudinal reinforcement in the bottom plates of beams BS1~BS3. The vertical axis represents the applied load (kN), while the horizontal axis indicates the strain (με) of the reinforcement.

Due to the beneficial effect of the prestressing tendons, the crack width of the bottom plate was obviously smaller than that of the web, and most of the measuring points of the longitudinal reinforcement in the bottom plate did not reach the tension yield strain when the specimen was damaged. Yet, upon comparing the stress increments in the bottom longitudinal reinforcement of beams BS1, BS2, and BS3, it was observed that most stress increments in BS3 were larger than those in BS1 and BS2. This difference arises because the continuous longitudinal reinforcement at the joint positions of BS1 and BS2 effectively absorbed the horizontal component of the principal tensile stress, thus distributing it among the bottom longitudinal reinforcements due to the principal tensile stress. In contrast, the web longitudinal reinforcement of BS3 was disconnected at the joint section, and the horizontal component of the principal tensile stress was primarily borne by the prestressing tendons in the bottom plate. This significantly influenced the tendons, leading to a notable increase in tensile stress. Consequently, the stress in the longitudinal reinforcement of the bottom plate increased. Therefore, it can be concluded that effective web longitudinal reinforcement mitigates axial tensile stress caused by shear, thereby significantly reducing the increase in tensile stress observed in the bottom longitudinal reinforcement due to shear.

### 5.3. Failure Mode

**Figure 19:**
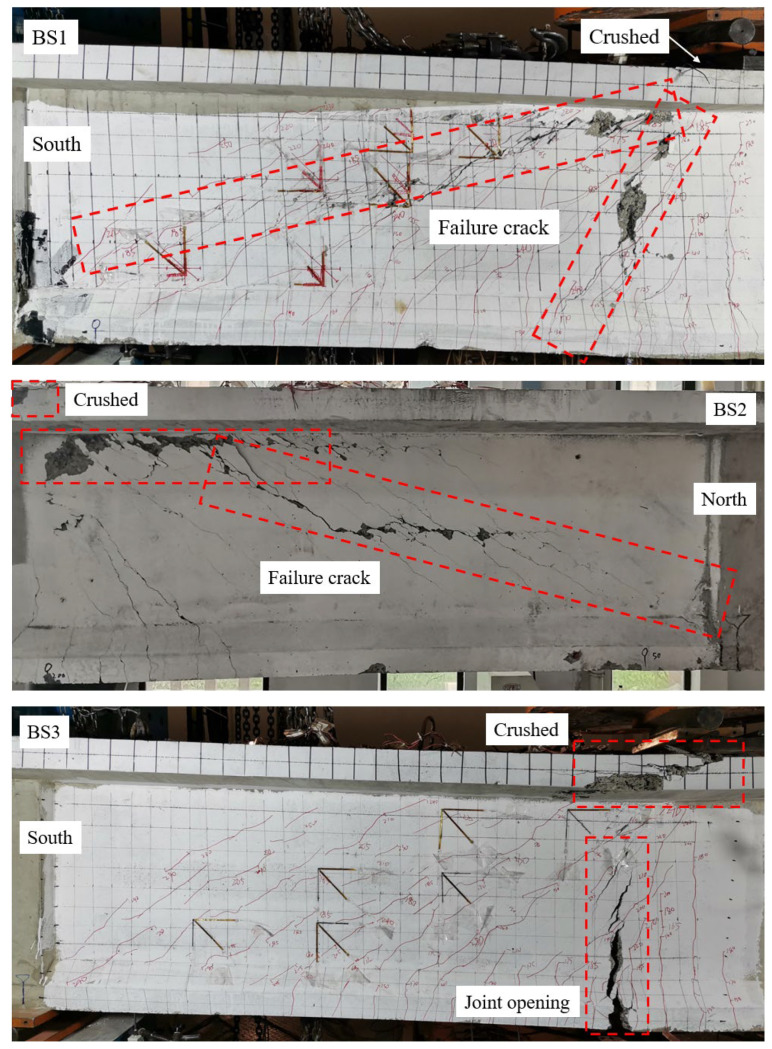
View of beams BS1~BS3 after failure.

Figure 19 shows the failure mode of beams BS1~BS3. When beam BS1 approached failure, two distinct types of cracks emerged in the web: thin web shear diagonal cracks. One of these cracks extended from the loading point to the support point, while the other spanned from the loading point to the main beam’s bottom edge, located 50 cm away from the loading point. Upon failure, these cracks widened significantly, causing spalling in the adjacent concrete. Additionally, the concrete in the compression zone near the loading point experienced local crushing.

As beam BS2 approached failure, a main crack gradually developed in the web, spanning from the loading point to the support point. Under continued loading, this main crack widened progressively, resulting in spalling of the web concrete and the interface concrete between the top plate and the web. Local crushing of the concrete occurred at the loading point, resembling the failure mode observed in beam BS1.

As beam BS3 was loaded, a primary crack progressively formed along the joint section (plain concrete). This crack extended toward the top plate, creating a corner in the mid-web region that pointed toward the loading point. With further loading, the concrete at the lower edge of the joint section started to yield, resulting in the gradual opening of the joint. This caused the neutral axis of the section to shift upward and the concrete compression zone to diminish, leading to stress redistribution within the joint. Eventually, the specimen displayed an inverted V-shaped opening along the joint, coupled with localized concrete crushing near the loading point, signaling impending failure. Key indicators of failure included plastic rotation of the segments on either side of the joint around the upper edge, as well as substantial deflection deformation of the specimen.

### 5.4. Deformation Characteristics

**Figure 20:**
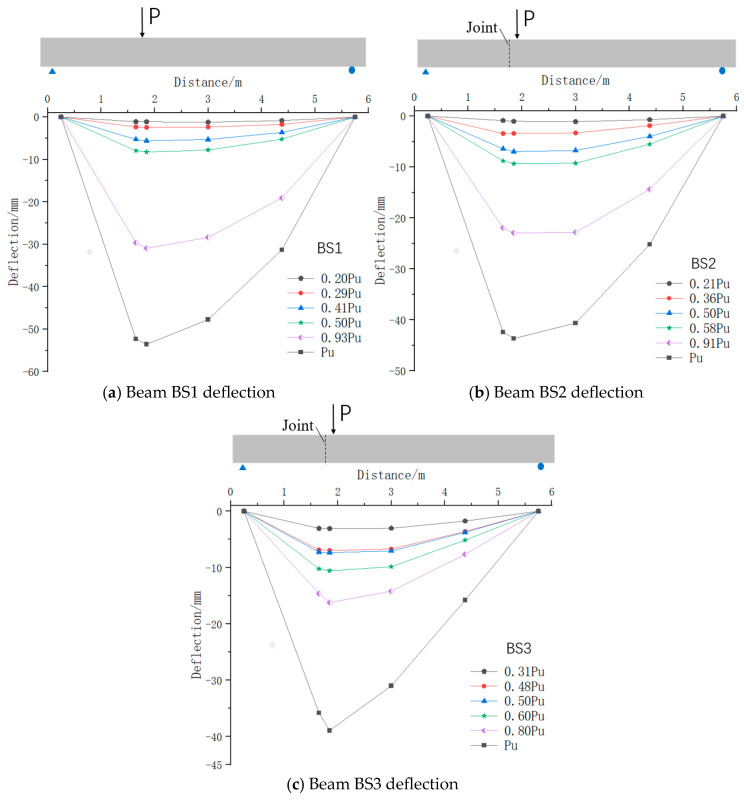
Specimen deflection development.

Figure 20 illustrates the vertical deflection distribution along the beam length at various load stages, such as the initial crack stage, incipient crack stage, mid-crack development stage, 1/2 ultimate load stage, complete crack development stage, and ultimate load stage. During the initial loading phase, beams BS1 to BS3 exhibited high structural stiffness, with all specimens undergoing integral deformation without cracking. As the load increased, cracks developed to varying extents in all specimens, resulting in a progressive reduction in structural stiffness. This led to a more significant vertical deflection near the loading point at the bottom edge. An inflection point gradually emerged at this location in the deflection curves of each specimen. At failure, the maximum deflection was observed at this position.

As cracks propagated uniformly and completely within the web of the shear span area of beams BS1 and BS2, the joint of BS2 remained closed. Vertical displacement development was consistent on both sides of the joint, with no vertical shear slip observed. Upon damage, beams BS1 and BS2 exhibited a deflection curve with a relatively gradual inflection point at the joint position, as shown in Figure 20a,b. After the primary crack formed in beam BS3, it progressively opened along the joint section, forming an inverted V-shaped gap. As this gap widened, differences in deflection due to specimen bending curvature appeared on either side of the joint. Upon failure of beam BS3, this deflection difference reached its maximum, accompanied by shear slip along the joint between segments. Consequently, the deflection curve of beam BS3 exhibited a distinct angular change at the joint, as illustrated in Figure 20c.

Furthermore, as depicted in Figure 20a–c, as the loading force increased, the cracking of beams BS1 and BS2 was accompanied by the progressive development of structural deflection. In other words, beams BS1 and BS2 accommodated the structural deformation by means of the increase in the number of cracks and the widening of the cracks. When the loading force was close to 0.91–0.93 Pu, the crack width and crack number of beams BS1 and BS2 were completely developed. The development of vertical deflection of beams BS1 and BS2 was mostly concentrated during the crack development. During the elastic phase, beam BS3 underwent uniform deformation, similar to beams BS1 and BS2. As the applied load approached 0.8 times the ultimate load (Pu), the number and width of cracks in BS3 became fully developed. The specimen accommodated structural bending primarily through the degree of joint opening. Vertical displacement in BS3 occurred mainly after the cracks were fully developed, corresponding to the formation of an inverted V-shaped opening at the joint. An observation can be made that the displacement increment of BS3 was significantly greater than that of beams BS1 and BS2 from the crack growing completely to the specimen failure.

### 5.5. Stiffness Analysis

**Figure 21:**
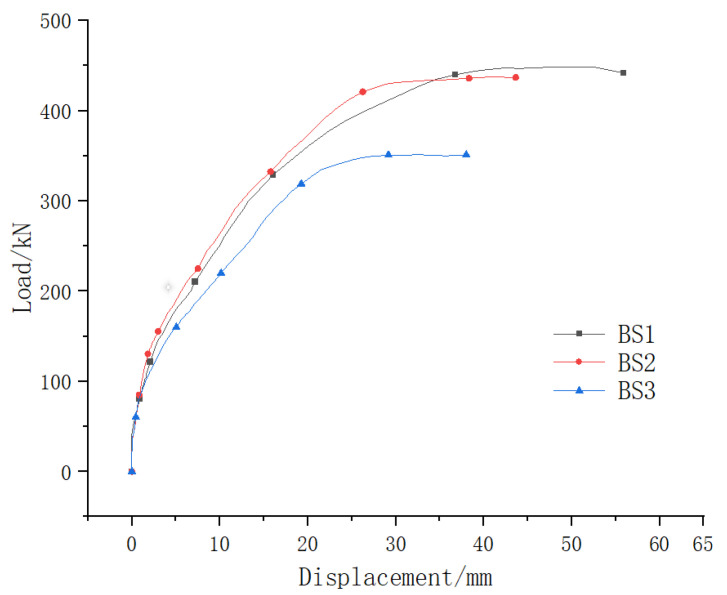
Load–displacement curves.

Figure 21 compares the load–displacement curves of beam BS1 (monolithic beam), beam BS2 (segmental beam with continuous longitudinal reinforcement), and beam BS3 (traditional segmental beam). The curves for beams BS1 and BS2 exhibited similar trends throughout the loading process, characterized by four distinct stages: elastic deformation, crack development, sustained loading, and eventual failure. In contrast, beam BS3 experienced an additional stage where the joint gradually opened after crack development, followed by sustained loading and failure. This joint opening in beam BS3 led to significant stress redistribution within the joint section. Specifically, the concrete tension zone became inactive, the neutral axis shifted upward, and the height of the concrete compression zone decreased progressively. These changes resulted in increased stress within the remaining concrete compression zone, causing it to enter the elastoplastic stage earlier than in beams BS1 and BS2. Consequently, the opened joint in beam BS3 significantly reduced structural stiffness.

The load–displacement curve development history of beams BS1 and BS2 surpassed that of beam BS3, demonstrating that continuous longitudinal reinforcement at the joints enhances the ductility of segmental beams. During the elastic stage, the load–displacement curves of all three beams were nearly identical. However, after cracking, beams BS1 and BS2 exhibited full crack development within the shear span area, with similar deformation progression. In contrast, cracks in beam BS3 were predominantly concentrated in the joint section, and its deformation progressed more rapidly compared to beams BS1 and BS2. This disparity highlights that continuous longitudinal reinforcement at the joint effectively restricts the width of the main crack, thereby improving the structural stiffness of segmental beams.

### 5.6. Shear Capacity

Table 8 presents the shear capacities of the tested specimens. The shear capacity of BS2 was slightly lower than that of BS1 by 5%, indicating a close performance between the two. In contrast, BS3 exhibited a 22.05% reduction in shear capacity compared to BS1. This significant difference can be attributed to the wider main crack at the joint in BS3, which caused an earlier upward shift of the neutral axis relative to beams BS1 and BS2. Consequently, the concrete compression zone in BS3 experienced a more substantial reduction, leading to a markedly lower failure load. These findings underscore the critical role of continuous longitudinal reinforcement at the joint in improving the shear strength of segmental beams.

## 6. Discussion

Table 9 presents the design methods of shear reinforcement according to major international specifications and the Grid Shear Reinforcement Theory.

(1) Stirrups only

At present, only the ACI [28] (American) specification and the GB [30] and JTG [31] (Chinese) specifications are widely adopted. Nonetheless, there is a significant disparity in how these specifications account for the shear contribution of concrete. The ACI specification considers concrete’s shear resistance as the load at which the first shear crack (whether flexural shear or web shear) appears. In contrast, the GB and JTG specifications define concrete’s shear resistance as the failure load of beams without web reinforcement, attributing a relatively higher shear contribution to concrete according to the Chinese standards. This discrepancy can lead to the potential underestimation of the required shear reinforcement in the Chinese specifications, as concrete is assumed to bear a substantial portion of the shear force until cracking occurs. The subsequent transfer of shear force to reinforcement upon web cracking can result in shear reinforcement yielding and disrupt the intended stress transfer path within the structure. In engineering practice, these differences may manifest as the development of wide diagonal cracks and ongoing downward deflection in prestressed concrete bridges.

(2) Stirrups and main longitudinal steel bars of upper and lower plates

The shear reinforcement theory, initially proposed by the University of Toronto and adopted by the American AASHTO [25] and European Codes [27], is rooted in truss models for practical applications. This theory establishes the structural equilibrium equation based on truss principles. In reinforced concrete structures, stirrups bear the vertical component of the principal tensile stress, while longitudinal steel bars at the upper and lower edges carry the horizontal component of the principal stress. Therefore, the main longitudinal steel bars not only fulfill flexural requirements but also accommodate stress increments due to shear forces. This mechanical balance allows longitudinal bars positioned at upper and lower edges to handle increasing horizontal components caused by diagonal crack development, supporting the overall structure without shear failure. However, the challenge lies in controlling the width of diagonal cracks, which stagger along the diagonal direction. Failure of the shear mechanism compromises normal stress transfer, including the prestressing effect, potentially leading to structural failure.

(3) Grid Shear Reinforcement Theory

The Grid Shear Reinforcement Theory [33,37] adopts a section method consistent with bending design principles. It emphasizes that when cracking occurs in any part of a concrete member due to principal tensile stress exceeding its strength, shear reinforcement composed of vertical and horizontal grid reinforcement must be implemented at corresponding positions along the section. This reinforcement is tasked with bearing both vertical and horizontal components of the principal stress, ensuring that none of the reinforcements yield at the limit stage. By maintaining all reinforcements in an unyielding state, this approach effectively controls diagonal cracks and preserves the shear stiffness of the structure. As such, it ensures the necessary transfer of normal stress without compromising structural integrity.

**Figure 22:**
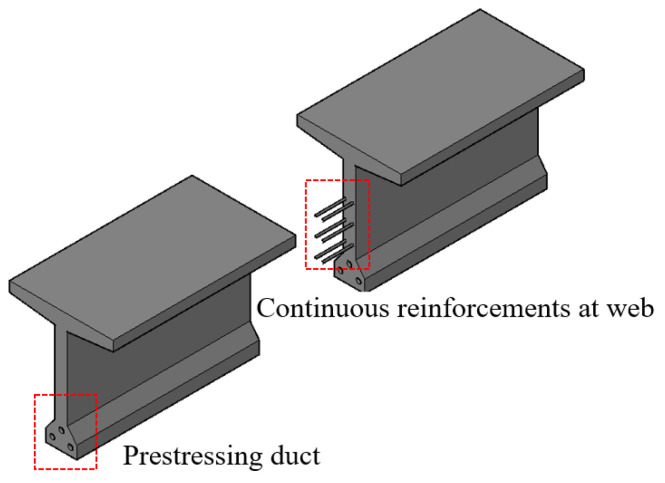
Longitudinally inserted reinforcements at the joint.

**Figure 23:**
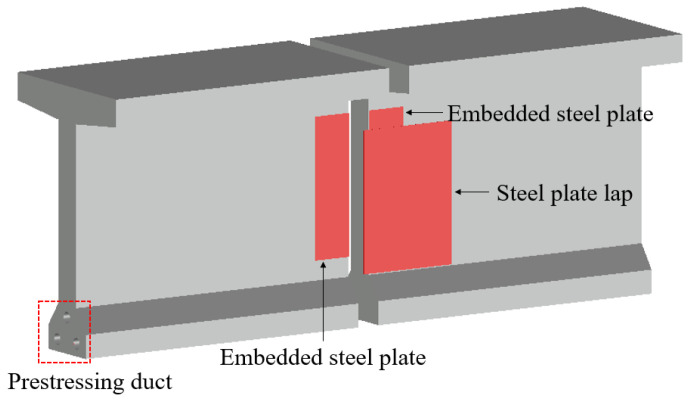
Gusset plate connecting.

The Grid Shear Reinforcement theory distinguishes shear rebars from traditional stirrups, recognizing that web longitudinal steel bars also function as shear rebars and must be calculated accordingly, not merely treated as “constructional reinforcements”. In structural joints, stirrups and longitudinal steel bars are discontinuous due to their placement. In practice, ensuring the “continuity” of stirrups is relatively straightforward—they are inherently spaced out, allowing joints to integrate seamlessly with the overall structure. The critical challenge lies in maintaining the continuity of longitudinal steel bars, which is essential for structural integrity and performance under shear forces. The internally prestressed tendons located at the top and bottom can be considered as continuous reinforcements in the structure. Therefore, ensuring the continuity of longitudinal reinforcements primarily focuses on the horizontal longitudinal steel bars within the height range of the web. Figure 22 shows the method of continuous longitudinal reinforcement by using bonded rebars in the web. Figure 23 shows the method of a gusset plate connecting in the web.

It should be noted that this study did not consider the impact of fire on the performance of joints. As highlighted by Bolina et al. [38], fire is a critical factor that must be accounted for in the design of concrete structures, as elevated temperatures can significantly degrade the mechanical properties of both concrete and reinforcement, thereby compromising the load-bearing capacity and safety of joints. Therefore, future research should further investigate the effects of fire on joint behavior to provide more comprehensive design guidelines. And under extreme loading conditions (e.g., seismic or impact loads), the behavior of joints with continuous reinforcement requires further investigation. The long-term durability of continuous longitudinal reinforcement, particularly in aggressive environments (e.g., exposure to chlorides or carbonation), needs to be evaluated. Potential issues such as corrosion of reinforcement and its impact on structural performance should be considered in future studies. Although continuous longitudinal reinforcement improves structural performance, it may increase construction complexity and cost compared to traditional methods. These factors should be carefully weighed in practical applications.

## 7. Conclusions

(1) The continuous longitudinal reinforcement at the joint effectively resisted the axial tension induced by shear forces, functioning similarly to stirrups in providing shear resistance. In contrast, the web longitudinal reinforcement near the joint in traditional segmental beams failed to reach the yield strain under tension, rendering it ineffective in fulfilling its intended shear role.
(2) The mechanical behavior of stirrups in segmental beams with continuous longitudinal reinforcement closely resembles that of monolithic beams. Specifically, stirrups near the joint effectively resisted the vertical component of the principal tensile stress during specimen failure, fully utilizing their shear reinforcement capacity. However, in traditional segmental beams with open joints, the absence of longitudinal reinforcement constraints led to the complete release of structural stress on both sides of the joint. Consequently, shear stress was transferred primarily through the prestressing tendons and the concrete shear compression zone at the upper edge of the joint, significantly diminishing the shear resistance provided by stirrups.
(3) In segmental beams with continuous longitudinal reinforcement, the web longitudinal reinforcement effectively bore the axial tension caused by shear, significantly reducing the tensile stress increase in the bottom longitudinal reinforcement. In contrast, traditional segmental beams, which feature discontinuous longitudinal reinforcement at the joint, exhibited a substantial increase in tensile stress in the bottom longitudinal reinforcement due to shear forces.
(4) The continuous longitudinal reinforcement in segmental beams effectively restrained the development of diagonal web cracks and limited crack widths, thereby enhancing structural stiffness. Furthermore, the load–displacement curves of these beams closely mirrored those of monolithic beams, demonstrating significantly higher structural stiffness and load-bearing capacity compared to traditional segmental beams.
(5) From the initiation of web cracking to specimen failure, the stress distribution near the joints of segmental beams with continuous longitudinal reinforcement largely adhered to the plane section assumption. This ensured that key mechanical behaviors—such as shear capacity, structural stiffness, crack propagation, and failure modes—were consistent with those observed in monolithic beams. The experimental results comprehensively validate the critical role of continuous longitudinal reinforcement at joint sections in segmental beams. This approach not only highlights its necessity but also establishes it as a vital method for mitigating the adverse effects of joints on mechanical performance.
(6) The findings of this study highlight the necessity of incorporating continuous longitudinal reinforcement in future design codes for precast concrete segmental beams (PCSBs).

## Figures and Tables

**Table 1:** Parameters of the test beams.

	Beams	BS1	BS2	BS3
Parameters				
Web width/mm		80	80	80
Beam height/mm		670	670	670
Effective height/mm		639	639	639
Stirrup spacing/mm		100	100	100
Shear span α/mm		1700	1700	1700
Shear span ratio/λ		2.66	2.66	2.66
Upper and lower longitudinal reinforcement ratio/%		0.614	0.614	0.614
Stirrup ratio/%		0.706	0.706	0.706
Longitudinal reinforcement type		Continuous at the joint	Continuous at the joint	Discontinuous at the joint
Web longitudinal reinforcement ratio/%		0.706	0.706	0.706
Erection		Monolithic	Segmental	Segmental
Load type		Shear-bending	Shear-bending	Shear-bending
Static scheme		Simply supported	Simply supported	Simply supported

**Table 2:** Properties of concrete.

Test Names	BS1	BS2	BS3
Strength/MPa	49	46	48

**Table 3:** Properties of reinforcement and prestressing tendon.

Specification and Dimension	C6	C8	C10	$\Phi^{S}15.2$
Yield strength/MPa	455.30	450.06	465.35	1658.5
Ultimate strength/MPa	617.78	625.88	658.35	1844.39
Elasticity modulus/MPa	2.00 × 10^5^	2.00 × 10^5^	2.00 × 10^5^	1.95 × 10^5^

**Table 4:** Effective prestress value of each specimen.

Beam	BS1	BS2	BS3
Effective prestressing/MPa	840	827	829

**Table 5:** Strain gage parameters.

Version	Electrical Resistance/Ω	Sensitivity Coefficient/%	Precision	Length × Width/mm
BX120-100AA	119.6 ± 0.1	2.08 ± 1	A	3 × 2

**Table 6:** Incipient crack in the bottom plate.

Beams	Loading Force /kN	Crack Type	Length /mm	Width /mm	Location	Cracks Sequence	Remark
BS1	90	Bending crack	20	0.050	Bottom edge of main beam at loading point	Initial crack	Incipient crack
	120	Bending crack	60	0.064	Bottom edge of main beam 60 cm away from loading point	Second crack	
	130	Bending crack	40	0.052	Bottom edge of main beam 40 and 50 cm away from loading point	Third crack	
BS2	95	Bending crack	30	0.060	Bottom edge of main beam at loading point	Initial crack	Incipient crack
	145	Bending crack	80	0.085	Bottom edge of joint	Second crack	
	150	Shear-Bending crack	100	0.075	Bottom edge of main beam 40 cm away from loading point	Third crack	
BS3	110	Bending crack	30	0.053	Bottom edge of main beam 10 cm away from loading point	Initial crack	Incipient crack
	150	Bending crack	30	0.060	Bottom edge of main beam at loading point	Second crack	
	155	Bending crack	50~70	0.073	Bottom edge of main beam within 30~60 cm from loading point	Third crack	

**Table 7:** Incipient cracks in the web.

Beams	Loading Force /kN	Crack Type	Length /mm	Width /mm	Location	Cracks Sequence	Remark
BS1	120	Diagonal shear crack in the web	200	0.072	At a distance of 50 cm from the loading point	Initial crack	Incipient crack
	125	Diagonal shear crack in the web	50	0.065	At a distance of 60 cm from the loading point	Second crack	
	130	Diagonal shear crack in the web	80	0.069	At a distance of 85 cm from the loading point	Third crack	
BS2	120	Diagonal shear crack in the web	80	0.073	At a distance of 35 cm from the loading point	Initial crack	Incipient crack
	155	Diagonal shear crack in the web	50	0.071	At a distance of 30 cm from the loading point	Second crack	
	160	Diagonal shear crack in the web	50~80	0.089	At distances of 20 cm and 60 cm from the loading point	Third crack	
BS3	130	Diagonal shear crack in the web	100	0.099	At a distance of 40 cm from the loading point	Initial crack	Incipient crack
	150	Diagonal shear crack in the web	100	0.179	At a distance of 45 cm from the loading point	Second crack	
	160	Diagonal shear crack in the web	200	0.079	At a distance of 20 cm from the loading point	Third crack	

**Table 8:** Test values of shear capacity.

Beam	BS1	BS2	BS3
Shear capacity/kN	449	435	350
VS BS1/%	1	−3.11	−22.05

**Table 9:** Shear reinforcement design methods according to main international specifications.

Main International Specifications	Shear Reinforcement	Shear Reinforcement
ACI [28], GB [30]/JTG [31]	Stirrups only	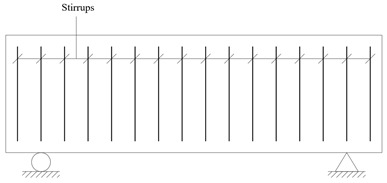
Eurocode [27], AASHTO [25,29]	Stirrups and main longitudinal steel bars of upper and lower plates	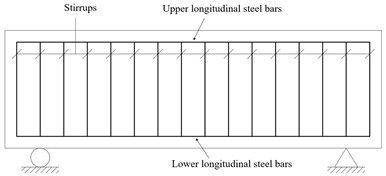
Grid Shear Reinforcement Theory [33,37]	Stirrups and all longitudinal steel bars fall within shear reinforcement	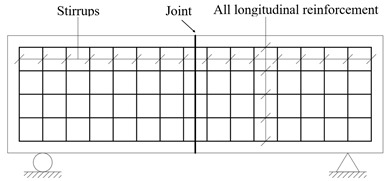

## Data Availability

The original contributions presented in this study are included in the article. Further inquiries can be directed to the corresponding author.

## References

[1] Zhan Y., Li Z., Chen Z., Shao J., Yue F., Liu F., Ma Z.J. (2022). Experimental and Numerical Investigations on Shear Performance of Key Tooth Joints of Precast Concrete Segmental Bridge Under Repeated Loading. Constr. Build. Mater..

[2] Choi J.S., Lee H.J., Yuan T.F., Yoon Y.S. (2023). Shear Strength of Steel Fiber Reinforced Lightweight Self-Consolidating Concrete Joints Under Monotonic and Cyclic Loading. Constr. Build. Mater..

[3] Luo Z., Wang Y., Wang T. (2022). Shear Behavior of Epoxy Joints in Precast Segmental Bridges Under Impact Loading. Eng. Struct..

[4] Rabbat B. (1987). Testing of Segmental Concrete Girders with External Tendons. PCI J..

[5] Macgregor R.J.F., Kreger M.E., Breen J.E. (1990). Strength and Ductility of a Three-Span Externally Post-Tensioned Segmental Box Girder Bridge Model. Earth Planet. Sci. Lett..

[6] Ramos G., Aparicio A.C. (1996). Ultimate Analysis of Monolithic and Segmental Externally Prestressed Concrete Bridges. J. Bridge Eng..

[7] Aparicio A.C., Ramos G., Casas J.R. (2002). Testing of Externally Prestressed Concrete Beams. Eng. Struct..

[8] Huang Z., Liu X. (2006). Modified Skew Bending Model for Segmental Bridge with Unbonded Tendons. J. Bridge Eng..

[9] Yuan A., Dai H., Sun D., Cai J. (2013). Behaviors of Segmental Concrete Box Beams with Internal Tendons and External Tendons Under Bending. Eng. Struct..

[10] Jiang H., Cao Q., Liu A., Wang T., Qiu Y. (2016). Flexural Behavior of Precast Concrete Segmental Beams with Hybrid Tendons and Dry Joints. Constr. Build. Mater..

[11] Jiang H., Li Y., Liu A., Ma Z.J., Chen L., Chen Y. (2018). Shear Behavior of Precast Concrete Segmental Beams with External Tendons. J. Bridge Eng..

[12] Moustafa S.E. (1974). Ultimate Load Test of a Segmentally Constructed Prestressed Concrete I-Beam. PCI J..

[13] Ramirez G., Macgregor R., Kreger M.E. Shear Strength of Segmental Structures. Proceedings of the Workshop AFPC External Prestressing in Structures.

[14] Turmo J., Ramos G., Aparicio A.C. (2005). FEM Study on the Structural Behaviour of Segmental Concrete Bridges with Unbonded Prestressing and Dry Joints: Simply Supported Bridges. Eng. Struct..

[15] Li G.P., Yang D.H., Lei Y. (2013). Combined Shear and Bending Behavior of Joints in Precast Concrete Segmental Beams with External Tendons. J. Bridge Eng..

[16] Brenkus N.R., Wagner D.J., Hamilton H.R. (2016). Experimental Evaluation of Shear Strength of an Innovative Splice for Prestressed Precast Concrete Girders. J. Bridge Eng..

[17] Takebayashi T., Deeprasertwong K., Leung Y.W.J. (2015). A Full-Scale Destructive Test of a Precast Segmental Box Girder Bridge with Dry Joints and External Tendons. Struct. Build..

[18] Hindi A., Macgregor R., Kreger M.E., Breen J.E. (1995). Enhancing Strength and Ductility of Post-Tensioned Segmental Box Girder Bridges. ACI Struct. J..

[19] Sivaleepunth C., Niwa J., Nguyen D.H., Hasegawa T., Hamada Y. (2009). Shear Carrying Capacity of Segmental Prestressed Concrete. Doboku Gakkai Ronbunshuu E.

[20] Halder R., Yuen T.Y.P., Chen W.-W., Zhou X., Deb T., Zhang H., Wen T.H. (2021). Tendon Stress Evaluation of Unbonded Post-Tensioned Concrete Segmental Bridges with Two-Variable Response Surfaces. Eng. Struct..

[21] Tran D.T., Pham T.M., Hao H., Chen W. (2021). Numerical Investigation of Flexural Behaviours of Precast Segmental Concrete Beams Internally Post-Tensioned with Unbonded FRP Tendons Under Monotonic Loading. Eng. Struct..

[22] Tran D.T., Pham T.M., Hao H., Chen W. (2021). Numerical Study on Bending Response of Precast Segmental Concrete Beams Externally Prestressed with FRP Tendons. Eng. Struct..

[23] Le T.D., Pham T.M., Hao H. (2020). Numerical Study on the Flexural Performance of Precast Segmental Concrete Beams with Unbonded Internal Steel Tendons. Constr. Build. Mater..

[24] Le T.D., Pham T.M., Hao H., Yuan C. (2019). Performance of Precast Segmental Concrete Beams Posttensioned with Carbon Fiber-Reinforced Polymer (CFRP) Tendons. Compos. Struct..

[25] American Association of State Highway and Transportation Officials (1999). Guide Specifications for Design and Construction of Segmental Concrete Bridges.

[26] (2004). Prestressing Concrete Structures with FRP Tendons.

[27] (2004). Eurocode 2: Design of Concrete Structures—Part 1–1. General Rules and Rules for Buildings.

[28] (2005). Building Code Requirements for Structural Concrete and Commentary.

[29] American Association of State Highway and Transportation Officials (2003). Load and Resistance Factor Design. Bridge Design Specifications.

[30] (2010). Code for Design of Concrete Structures.

[31] (2018). Specifications for Design of Highway Reinforced Concrete and Prestressed Concrete Bridges and Culverts.

[32] Xu D., Zhang Y., Liu C., Turmo J. (2013). Shear Design of Concrete Beams Reinforced with Grid Reinforcement. Mag. Concr. Res..

[33] Sun Y., Xu D., Chen B., Xu F.Y., Zhu H.P. (2018). Three-Dimensional Reinforcement Design Method and Program Realization for Prestressed Concrete Box-Girder Bridges Based on a Specific Spatial Lattice Grid Model. Eng. Struct..

[34] Zou Y., Xu D., Duanmu X. (2024). Tests and Calculation Methods for the Shear Performance of Steel Shear Keyed Joint Segment Beams. Arch. Civ. Mech. Eng..

[35] Jiang H., Hu Z., Cao Z., Gao X., Tian Y., Sun X. (2022). Experimental and Numerical Study on Shear Performance of Externally Prestressed Precast UHPC Segmental Beams Without Stirrups. Structures.

[36] Zou Y., Xu D. (2024). Experimental Study on the Bending Behavior of Precast Concrete Segmental Bridges with Continuous Rebars at Joints. Buildings.

[37] Xu D., Zhang Y., Xu F., Gauvreau P. (2017). Unified Flexural Design Method for Deep and Shallow Beams Using Non-Linear Grid Model. Struct. Eng. Int..

[38] Bolina F.L., Lago B.D., Rodríguez E.D. (2024). Effects of Thermal Properties on Temperature Field of UHPC Structures Under Fire Conditions. Constr. Build. Mater..

